# CPAP enhances and maintains chronic inflammation in hepatocytes to promote hepatocarcinogenesis

**DOI:** 10.1038/s41419-021-04295-2

**Published:** 2021-10-22

**Authors:** Ruo-Yu Chen, Chia-Jui Yen, Yih-Jyh Lin, Ju-Ming Wang, Ting-Fen Tasi, Yu-Chuan Huang, Yao-Wen Liu, Hung-Wen Tsai, Ming-Hao Lee, Liang-Yi Hung

**Affiliations:** 1 Department of Biotechnology and Bioindustry Sciences, College of Bioscience and Biotechnology, National Cheng Kung University, Tainan, Taiwan; 2 Department of Oncology, Tainan, Taiwan; 3 Department of Surgery, National Cheng Kung University Hospital, College of Medicine, National Cheng Kung University, Tainan, Taiwan; 4 Department of Life Sciences and Institute of Genome Sciences, National Yang Ming Chiao Tung University, Taipei, Taiwan; 5 Genomics Research Center, Academia Sinica, Taipei, Taiwan; 6 Department of Clinical Pathology, Kuo General Hospital, Tainan, Taiwan; 7 Department of Pathology, National Cheng Kung University Hospital, College of Medicine, National Cheng Kung University, Tainan, Taiwan; 8 Department of Pharmacology, College of Medicine, National Cheng Kung University, Tainan, Taiwan; 9 Institute for Cancer Biology and Drug Discovery, College of Medical Science and Technology, Taipei Medical University, Taipei, Taiwan; 10 Graduate Institute of Medicine, College of Medicine, Kaohsiung Medical University, Kaohsiung, Taiwan; 11 University Center for Bioscience and Biotechnology, National Cheng-Kung University, Tainan, Taiwan

**Keywords:** Liver cancer, Tumour immunology

## Abstract

Chronic and persistent inflammation is a well-known carcinogenesis promoter. Hepatocellular carcinoma (HCC) is one of the most common inflammation-associated cancers; most HCCs arise in the setting of chronic inflammation and hepatic injury. Both NF-κB and STAT3 are important regulators of inflammation. Centrosomal P4.1-associated protein (CPAP), a centrosomal protein that participates primarily in centrosome functions, is overexpressed in HCC and can increase TNF-α-mediated NF-κB activation and IL-6-induced STAT3 activation. A transgenic (Tg) mouse model with hepatocyte-specific CPAP expression was established to investigate the physiological role of CPAP in hepatocarcinogenesis. Obvious inflammatory cell accumulation and fatty change were observed in the livers of *CPAP* Tg mice. The alanine aminotransferase (ALT) level and the expression levels of inflammatory genes, such as IL-6, IL-1β and TNF-α, were higher in *CPAP* Tg mice than in wild type (WT) mice. High-dose/short-term treatment with diethylnitrosamine (DEN) increased the ALT level, proinflammatory gene expression levels, and STAT3 and NF-κB activation in *CPAP* Tg mice; low-dose/long-term DEN treatment induced more severe liver tumor formation in *CPAP* Tg mice than in WT mice. CPAP can increase the expression of chemokine (C-C motif) ligand 16 (CCL-16), an important chemotactic cytokine, in human hepatocytes. CCL-16 expression is positively correlated with *CPAP* and *TNF-α* mRNA expression in the peritumoral part of HCC. In summary, these results suggest that CPAP may promote hepatocarcinogenesis through enhancing the inflammation pathway via increasing the expression of CCL-16.

## Introduction

Hepatocellular carcinoma (HCC) is one of the most aggressive cancers and the second leading cause of cancer mortality worldwide [1]. Approximately 70–90% of HCC cases are associated with chronic inflammation which may result from hepatitis infection, alcoholic liver disease, or nonalcoholic fatty liver disease [2]. During liver inflammation, various types of cells interact with chemokines to prevent the spread of pathogen infection; and these interactions maintain physiological homeostasis through a negative feedback mechanism [3, 4]. However, the uncontrolled action of regulatory mechanisms resulting from sustained inflammation (e.g., macrophage polarization toward the M2 phenotype, increased immunosuppressive cell infiltration, and prolonged or excessive inflammatory responses) will cause nonresolving inflammation and subsequently lead to cellular oncogenic transformation as well as genetic and epigenetic changes [4, 5]. In addition, HCC risk factors usually lead to dysregulated production of pro- and inflammatory soluble factors from immune cells, epithelial cells and endothelial cells; therefore, HCC has been considered a typical nonresolving inflammatory disease [4]. Recently, many studies have investigated whether the regulatory relationship between various proinflammatory cells (including immune cells) and inflammatory mediators within the tumor microenvironment is a potent target for cancer prevention and therapy [6–8].

In chronic hepatitis, both hyperactivation of NF-κB and STAT3 in immune cells and hepatocyte injury contribute to the production of pro- and inflammatory factors that maintain a protumor microenvironment in the liver [9]. Although making a definitive diagnosis of liver cancer by hyperactivation of NF-κB and STAT3 is difficult, to date, the efficacy of several developed drugs targeting specific cytokines, such as IL-6, to inhibit NF-κB and STAT3 hyperactivation has been evaluated in phase I and phase II clinical trials in various cancer types [10–12].

Chemokine (C-C motif) ligand 16 (CCL-16), also called liver-expressed chemokine (LEC), is strongly expressed by liver parenchymal cells and also expressed by the thymus and spleen [13, 14]. Biological functions of CCL-16 have been demonstrated to be involved in the immune response and chemotaxis by binding with CCR1, CCR2, CCR5 and CCR8 [14–17]. Several lines of evidence have indicated that CCL-16 plays an important role in immune responses as well as inflammatory activity. CCL-16 can drive the chemotaxis of monocytes, lymphocytes, eosinophils and dendritic cells; [18–20] CCL-16 and IL-10 can cooperatively enhance monocyte infiltration [21]. Importantly, CCL-16 can increase the effector and antigen‐presenting functions of macrophages and augment T cell cytotoxicity [22]. High levels of CCL-16 in serum and plasma are closely correlated with inflammation-related diseases, such as irritable bowel syndrome (IBS) and ulcerative colitis (UC) [23–25]. CCL-16 can regulate angiogenesis by interacting with CCR1 in endothelial cells; and the restricted expression of CCL-16 in liver cells may therefore contribute to hepatic vasculogenesis during hepatocarcinogenesis [26]. However, the role and regulatory mechanism of CCL-16 in HCC remain unclear.

Centrosomal P4.1-associated protein (CPAP) is a component of the γ-tubulin complex and participates in centrosome-related functions [27, 28]. Our previous studies demonstrated that CPAP is required for the activation of TNF-α-mediated NF-κB and IL-6-mediated STAT3 signaling in HCC [29–31]. SUMO-1-modified CPAP enhances the transcriptional activity of NF-κB in HCC cells [31]; overexpressed CPAP directly interacts with STAT3 to enhance IL-6-mediated STAT3 activation and promote HCC tumor growth and metastasis [29]. However, the underlying mechanisms and cross‐talk between CPAP and chronic inflammation in the liver are still unclear. Here, we observed several pathological features of inflammation in transgenic (Tg) mice with liver-specific CPAP overexpression. In a model of diethylnitrosamine (DEN)-induced hepatic injury in *CPAP* Tg mice, ALT levels, apoptosis, and the tumor number and size were increased. In hepatocytes, SUMO-1 modification maintained CPAP protein stability upon IL-6 or TNF-α stimulation. SUMOylated CPAP and TNF-α form an inflammatory feedback loop to accelerate hepatocarcinogenesis. Furthermore, CPAP overexpression promoted proinflammatory cell infiltration through TNF-α/CCL-16 signaling. Our studies provide evidence for the critical role of CPAP in inflammation-mediated hepatocarcinogenesis, and CPAP-mediated upregulation of CCL-16 can be a serum biomarker for early diagnosis of premalignant lesions of HCC.

## Results

### Hepatocyte overexpressed CPAP promotes chronic inflammation and induces HCC tumorigenesis

**Fig. 1:**
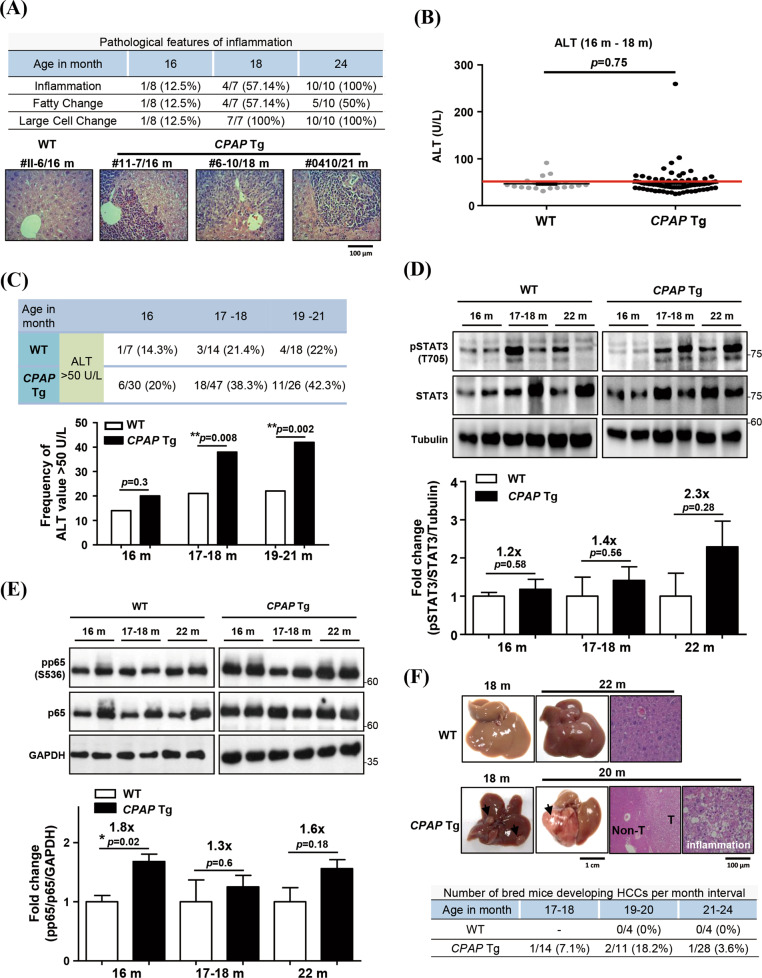
*CPAP* Tg mice display inflammatory features and promotion of hepatic tumorigenesis. **A** (Upper) The histopathological features of inflammation, including inflammatory cell infiltration, fatty changes and large cell change, in *CPAP* Tg and WT mice of various ages. (Lower) Liver tissues of *CPAP* Tg and WT mice were collected for H&E staining. Four representative liver tissues from one 16-month-old WT (#11-6/16 m) and three *CPAP* Tg (#11-7/16 m, #6-10/18 m, and #0410/21 m) are shown. **B** Serum ALT levels in *CPAP* Tg mice (*n* = 77) and WT mice (*n* = 21) aged 16 months (16 m) to 18 months (18 m) were measured with a FUJIFILM DRI-CHEM 4000i chemistry analyzer. Quantitative results are shown as the mean ± SEM values. Student’s t test, *p* = 0.75. **C** Percentages of *CPAP* Tg and WT mice with a serum ALT level higher than 50 U/L. The quantitative results of the frequency of ALT > 50 U/L were analyzed by chi-square test. **p < 0.01. **D**, **E** Total lysates of liver tissues from WT and *CPAP* Tg mice with different ages were collected for WB analysis using the indicated antibodies. Livers were collected form two mice of each age for analysis. Tubulin and GAPDH were used as loading controls. Quantitative results of the WB analysis are shown below. **F** Representative images of gross liver tumors and H&E-stained sections from WT and *CPAP* Tg mice of different ages are shown. Inflammation, tumor (T) tissues and nontumor (Non-T) tissues are shown. The incidence rates of liver tumors in WT and *CPAP* Tg mice from 17 to 24 months of age are listed in the table.

CPAP was reported to be an essential factor for TNF-α-mediated NF-κB and IL-6-mediated STAT3 activation in HCC [29–31]. Upon HBV infection, the viral oncoprotein HBx transcriptionally increases the expression of CPAP to enhance HCC development [30]. To evaluate the effects of CPAP in hepatocarcinogenesis, we assessed the physiological roles of overexpressed CPAP in hepatocytes. We generated Tg mice expressing human *CPAP* under the control of the human albumin promoter with two copies of chicken β-globin 5’HS4 as insulators (for details, please see the Materials and Methods section). Founder mice were generated by injecting the albumin-driven *CPAP* construct into fertilized eggs of C57BL/6 mice (Supplementary Fig. S1A); three lines of liver-specific *CPAP* Tg mice were selected for further investigation. Genotyping of *CPAP* Tg mice was performed by PCR (Supplementary Fig. S1B), and *CPAP* Tg mice were phenotypically similar to wild type (WT) mice. At 16 months of age, pathological features of inflammation, including inflammatory cell infiltration, fatty changes and liver cell dysplasia (large cell change), appeared in the livers of *CPAP* Tg mice. Inflammatory cell infiltration and liver cell dysplasia, appeared in all *CPAP* Tg mice (10/10) at 24 months of age (Fig. 1A). The serum ALT levels did not differ significantly between WT and *CPAP* Tg mice aged 16 months to 18 months (Fig. 1B). We analyzed the serum ALT level in mice at different ages and found that the percentage of mice with a serum ALT level > 50 U/L was higher in the *CPAP* Tg groups than in the WT groups aged 17 months to 18 months and 19 months to 21 months (Fig. 1C and Supplementary Fig. S2). Increased NF-κB and STAT3 activity was observed in the livers of *CPAP* Tg mice aged 16 months to 22 months (Fig. 1D, E). Importantly, a certain percentage of *CPAP* Tg mice exhibited HCC from approximately 17 months of age (Fig. 1F). These results suggested that hepatic overexpression of CPAP may cause HCC formation via the sustained chronic inflammation.

### CPAP overexpression enhances DEN-induced inflammation and liver tumor growth

**Fig. 2:**
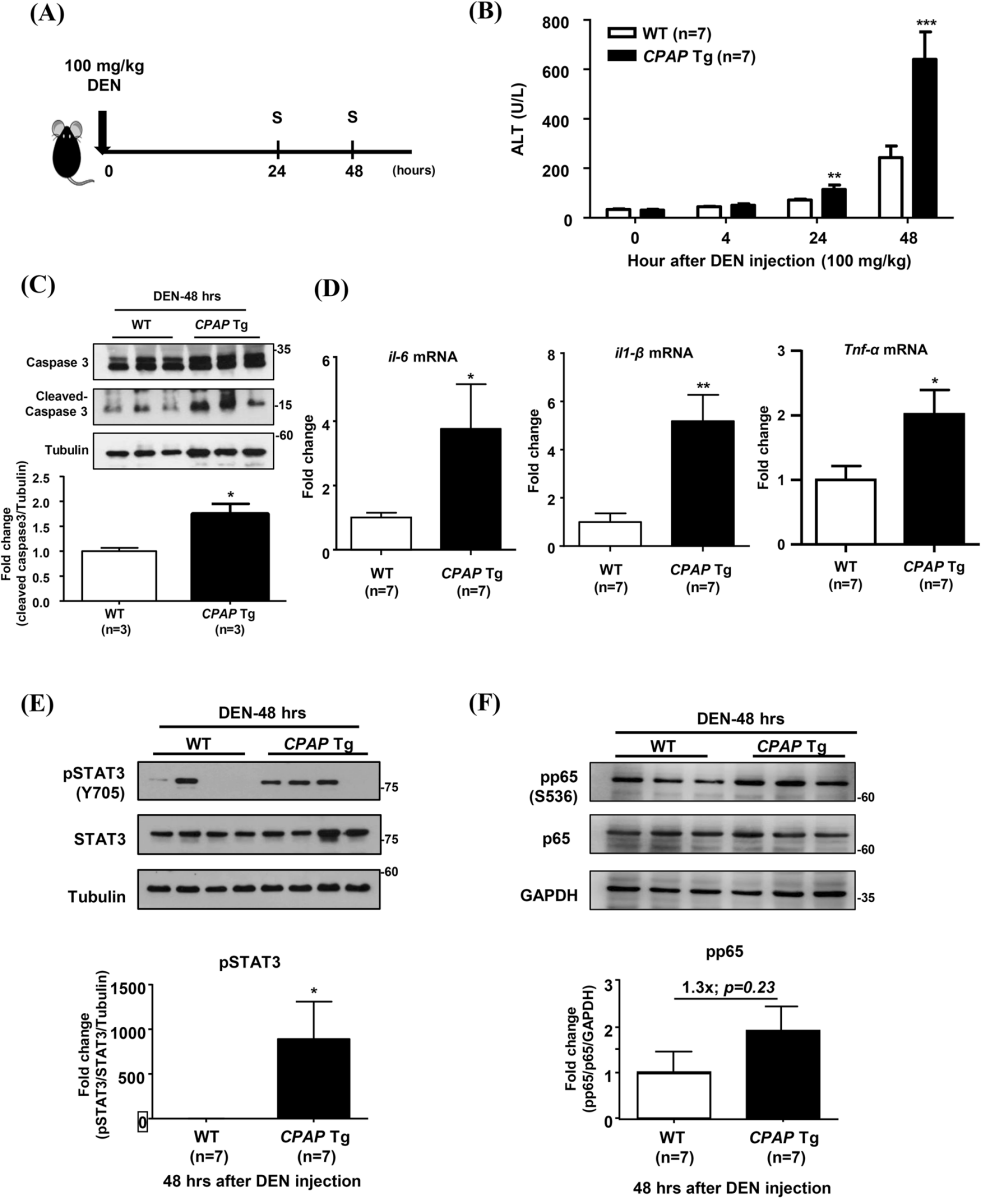
CPAP enhances DEN-induced inflammation and liver damage. **A** Schematic diagram of the protocol for high-dose DEN treatment in *CPAP* Tg and WT mice. Two‐month‐old male mice were injected IP with 100 mg/kg DEN (arrow). At 24 h and 48 h, mice were sacrificed (S), and liver tissue was collected for analysis. **B** The serum ALT levels in *CPAP* Tg mice (n = 7) and WT mice (n = 7) were measured 0, 4, 24 and 48 h after DEN injection. **C** Forty-eight hours after DEN treatment, liver tissues were collected from *CPAP* Tg and WT mice to assess apoptosis by WB analysis using anti-caspase 3 and anti-cleaved caspase 3 antibodies. Quantitative results are shown below. Tubulin was used as the loading control. **D** RT-qPCR analysis of proinflammatory factor expression levels in the liver tissues of *CPAP* Tg and WT mice 48 h after DEN injection. The data are presented as the mean ± SEM values. *p < 0.05, **p < 0.01. **E**, **F** Activation of STAT3 and NF-κB in the livers of DEN-treated mice was evaluated by WB analysis using anti-phospho-STAT3/Y705 (**E**) and anti-phosph-p65/S536 antibodies (**F**). WB analysis was performed on liver tissues from three representative *CPAP* Tg and WT mice, and quantitative results from seven mouse livers are shown below. Tubulin and GAPDH were used as loading controls.

**Fig. 3:**
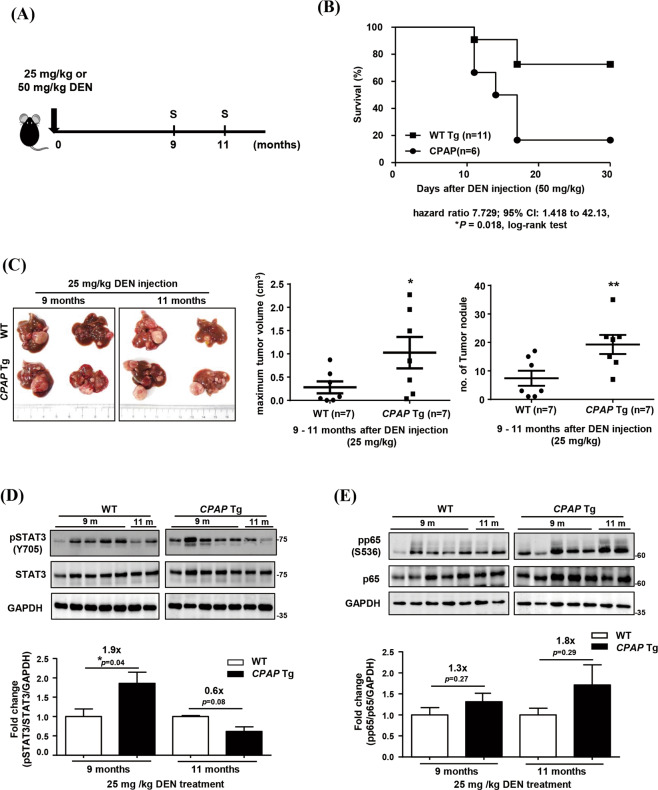
CPAP enhances DEN-induced hepatic tumorigenesis. **A** Schematic illustration of the protocol for low-dose DEN (25 mg/kg or 50 mg/kg) treatment in *CPAP* Tg and WT mice (arrow). Mice were sacrificed 9 and 11 months after DEN injection (S). **B** Survival rate of *CPAP* Tg (*n* = 6) and WT mice (*n* = 11) after 50 mg/kg DEN injection. Statistical analysis was performed using the log-rank test (Mantel-Cox method). HR = 7.729; 95% CI: 1.418 to 42.13, *p* = 0.018. **C** Representative gross images of liver tumors in WT and *CPAP* Tg mice 9 and 11 months after 25 mg/kg DEN injection are shown (left); the tumor volume (middle) and nodule number (left) were determined. **D**, **E** Activation of STAT3 (**D**) and NF-κB (**E**) in liver tissues of DEN-treated mice was evaluated as described in the legend for Fig. 2E, F. GAPDH was used as the loading control, and the quantitative results are shown. *p < 0.05.

Previous reports indicated that the pathologic and genetic alterations seen in DEN-induced carcinogenic hepatic injury are similar to those seen in human HCC [32, 33]. To further investigate the effects of CPAP on inflammation-induced HCC formation, *CPAP* Tg mice were treated with DEN to induce hepatic injury and hepatocarcinogenesis. High-dose/short-term and low-dose/long-term DEN treatments were administered to *CPAP* Tg and WT mice (Figs. 2A and 3A). After 24 h and 48 h of high-dose DEN (100 mg/kg) treatment, the serum ALT level in *CPAP* Tg mice was higher than that in WT mice (Fig. 2B). Nonresolving inflammation has been reported to increase DNA damage and cytokine-induced compensatory cell proliferation, both of which promote tumor growth [4]. The liver tissues of high-dose DEN-treated *CPAP* Tg mice showed increased levels of cleaved-caspase 3 protein and *IL-1β*, *IL-6* and *TNF-α* mRNAs (Fig. 2C, D), as well as increased activation of STAT3 and NF-κB (Fig. 2E, F). For the low-dose/long-term DEN treatment (Fig. 3A), we found that a poor survival rate was observed in *CPAP* Tg mice treated with 50 mg/kg DEN (Fig. 3B). In order to determine the effect of CPAP in liver tissues within a chronic inflammatory environment, *CPAP* Tg and WT mice were treated with a lower dosage of DEN (25 mg/kg) for 9–11 months. The results showed that low-dose DEN (25 mg/kg) treatment resulted in a higher tumor volume and number of tumor nodules in *CPAP* Tg mice than WT mice mice (Fig. 3C). As expected, activation of STAT3 and NF-κB was increased in livers of those low-dose and long-term DEN-treated *CPAP* Tg mice (Fig. 3D, E). These results suggested that overexpression of CPAP enhances inflammation-induced hepatocarcinogenesis through increased activation of STAT3 and NF-κB.

Previous studies reported that HBV-induced chronic liver inflammation is considered a major causative factor of hepatocarcinogenesis [4, 34]. CPAP increases HBx protein stability in an NF-κB-dependent manner, and facilitates HCC growth and progression [30]. To further investigate the ability of overexpressed CPAP in inflammation-induced hepatocarcinogenesis, we generated *CH* Tg mice. Liver tumors were observed in 100% (7/7) of *CH* Tg mice between 10 and 14 months of age, and only 60% (6/10) of *HBx* Tg at that same age (Supplementary Fig. S3). These results confirmed that CPAP overexpression enhances chronic inflammation-mediated hepatocarcinogenesis.

### SUMO-1 modification maintains CPAP protein stability

**Fig. 4:**
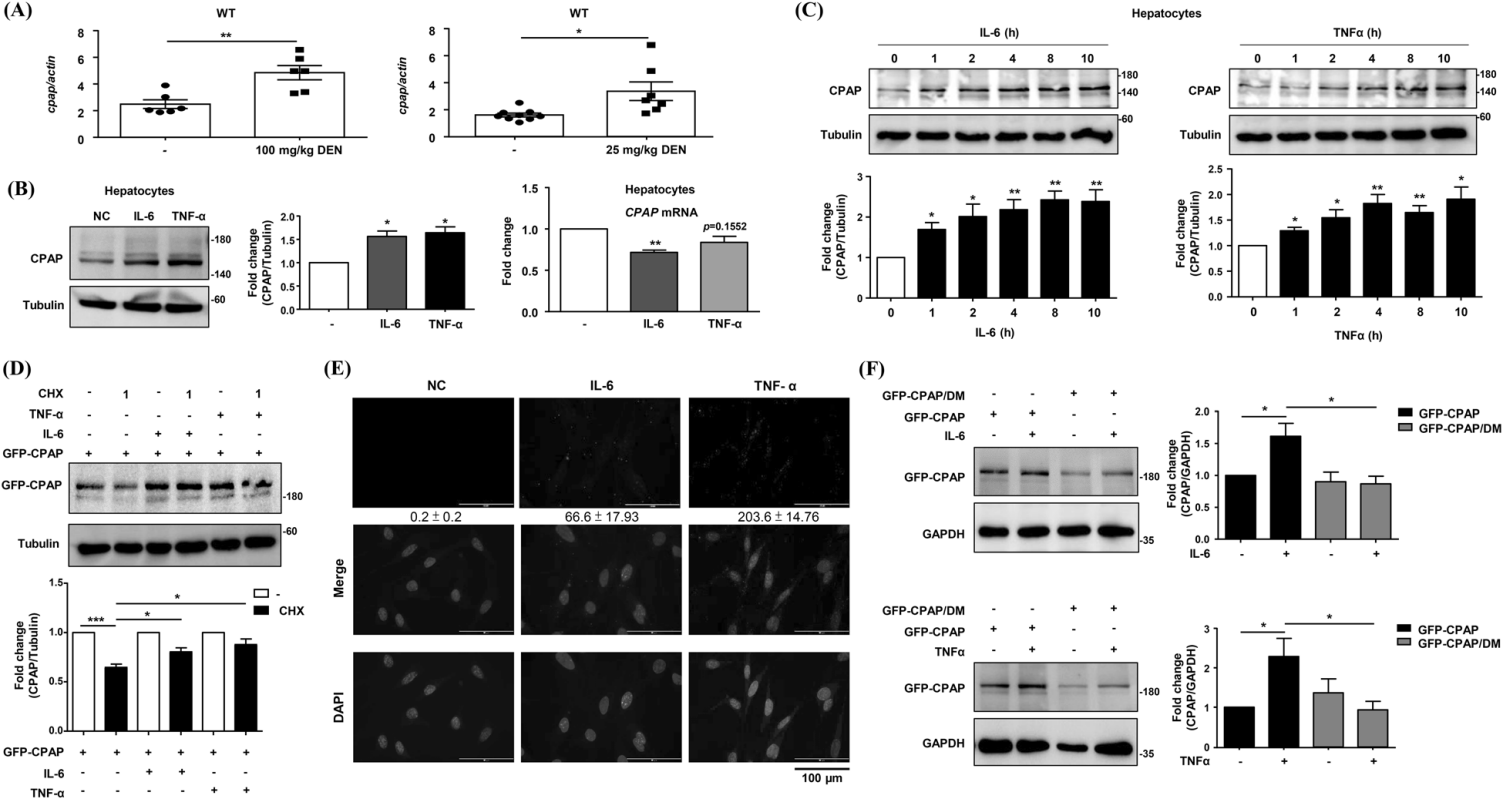
Treatment with either IL-6 or TNF-α maintains CPAP protein stability via SUMO-1 modification. **A** Expression levels of *cpap* mRNA in liver tissues from WT mice treated with 100 mg/kg DEN (left, *n* = 6) for 48 h and with 25 mg/kg DEN (left, *n* = 7) for 11 months were determined by RT-qPCR and normalized to *actin* mRNA. Liver tissues from untreated mice (-) were used as the negative control. **B** Human hepatocytes treated with 25 ng/ml IL-6 or 10 ng/ml TNF-α for 24 h were collected for WB analysis (left) and RT-qPCR (right). Tubulin was used as the loading control for WB analysis; the expression level of *CPAP* mRNA was normalized by *actin* mRNA. **C** Human hepatocytes were treated with 25 ng/ml IL-6 (upper) or 10 ng/ml TNF-α (lower) for the indicated time periods. Total cell lysates were collected and subjected to WB analysis. Tubulin was used as the loading control. The quantitative results are shown. **D** Human hepatocytes were transiently transfected with GFP-CPAP and treated with 25 ng/ml IL-6 or 10 ng/ml TNF-α for 4 h and then with 200 μg/ml cycloheximide (CHX) for an additional 1 h. Total cell lysates were collected for WB analysis using anti-GFP antibodies. Tubulin was used as the loading control. The quantitative results are shown. **E** In situ PLA was performed in IL-6 or TNF-α-treated human hepatocytes using anti-CPAP and anti-SUMO-1 antibodies. PLA signals were detected by fluorescence microscopy as distinct bright red puncta at 400X magnification. **F** Human hepatocytes transfected with GFP-CPAP or GFP-CPAP SUMO-deficient mutant (GFP-CPAP/DM) were treated with (+) or without (-) TNF-α (left) or IL-6 (right) for 8 h. Total cell lysates were collected and subjected to WB analysis using anti-GFP antibodies. GAPDH was used as the loading control. The quantitative results are shown.

Our results suggested that CPAP plays an important role in liver inflammation (Figs. 1D,E,2E,F and 3D,E; [29, 31]). Here, we evaluated the expression level of *CPAP* mRNA in the livers of DEN-treated mice. The results showed that *CPAP* mRNA was increased in the livers of DEN-treated WT mice (Fig. 4A). Since DEN treatment results in enhanced hepatic inflammation (2E-2F, and 3D-3E), we speculated that the increased transcription of *CPAP* may be due to hyperactivated STAT3 and NF-κB signaling in the livers of DEN-treated mice. To investigate this possibility, we measured the expression level of CPAP in hepatocytes under IL-6 or TNFα treatment. Unexpectedly, only the protein expression level, not the mRNA expression level, of CPAP was increased in human hepatocytes upon IL-6 or TNF-α stimulation (Fig. 4B). The protein expression of CPAP gradually increased with cytokine treatment (Fig. 4C), but the *CPAP* mRNA level did not change (Supplementary Fig. S4).

The literature indicates that SUMO-1 modification plays a crucial role in hepatitis virus replication and is also associated with inflammatory liver diseases and HCC development [35–37]. In addition, our previous report indicated that CPAP can be SUMO-1 modified upon cytokine stimulation [31]. Therefore, we investigated CPAP protein stability under IL-6 or TNF-α treatment. The results indicated that CPAP protein stability was increased in hepatocytes upon IL-6 or TNF-α treatment (Fig. 4D), and the in situ PLA results indicated that CPAP is SUMO-1 modified in hepatocytes after the addition of IL-6 or TNF-α (Fig. 4E). In contrast, the level of SUMO-1-deficient CPAP [31] was not increased in hepatocytes upon IL-6 or TNF-α treatment (Fig. 4F). These results suggested that SUMO-1 modification increases CPAP protein stability in chronically inflamed hepatocytes.

### CPAP maintains the inflammatory status in HCC-adjacent normal tissues (NT) by increasing STAT3 and NF-κB activity

**Fig. 5:**
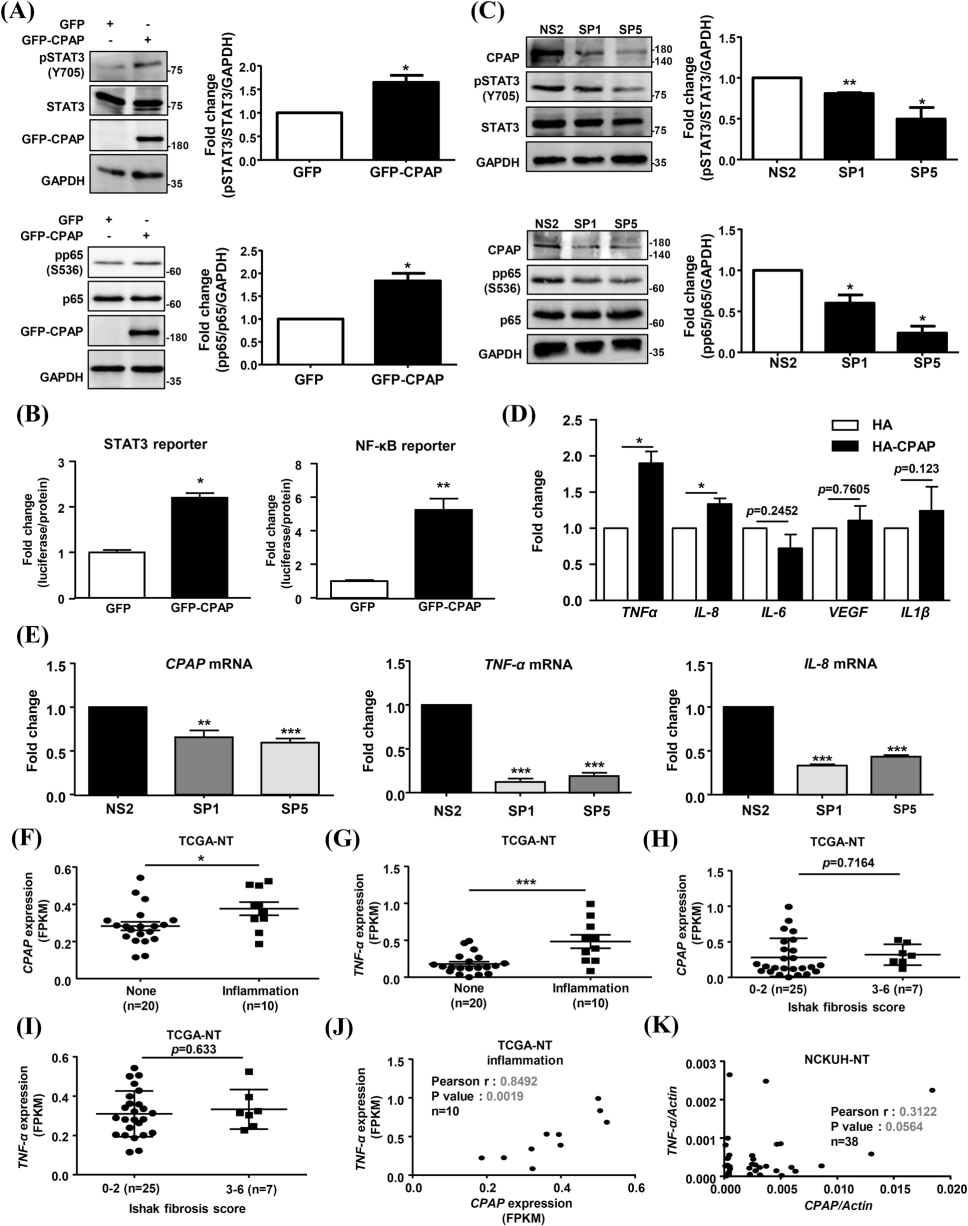
CPAP overexpression positively correlates with inflammation in nontumorous part of HCCs via enhanced STAT3 and NF-κB activation. **A**, **B** Human hepatocytes were transfected with GFP and GFP-CPAP, and then WB analysis was then performed using the indicated antibodies (**A**). The quantitative results are shown. The transcriptional activity of STAT3 and NF-κB was assessed by a reporter assay in GFP and GFP-CPAP-expressing cells (**B**). **C** Human hepatocytes transfected with *CPAP* shRNA, pSUPER-SP1 (SP1) and pSUPER-SP5 (SP5) [29], were collected for WB analysis as described above. pSUPER-NS2 (NS2) is the siRNA vector control. The quantitative results are shown. **D**, **E** The mRNA expression levels in hepatocytes transfected with HA-CPAP (**D**) and *CPAP* shRNA (SP1 and SP5) (**E**) were analyzed by RT-qPCR. **F**–**I** *CPAP* (F and H) and *TNF-α* (G and I) mRNA expression levels in adjacent normal liver tissues (NT) from HCC patients based on the lymphocytic inflammation status (**F**–**G**: normal, *n* = 20, inflammation, *n* = 10) and the Ishak fibrosis score (**H**–**I**: *n* = 32; 0: no fibrosis, 1: portal fibrosis (some), 2: portal fibrosis (most), 3: bridging fibrosis (occasional), 4: bridging fibrosis (marked), 5: incomplete cirrhosis, 6: cirrhosis) in the TCGA-LIHC dataset. The gene expression levels were normalized by FPKM normalization. **J**, **K** Correlation of *CPAP* and *TNF-α* mRNA levels in adjacent normal liver tissues (NT) with lymphocytic inflammation from TCGA dataset (**J**; *n* = 10) and the NCKUH HCC cohort (**K**; *n* = 38, Supplementary Table 1) was identified by the Pearson correlation coefficient. The data are presented as the mean ± SEM values. *p < 0.05, **p < 0.01. Information of the Ishak fibrosis scores (0-6) and adjacent normal tissue inflammation grades (lymphocytic infiltration: slight, *n* = 20; mild+severe, *n* = 10) in the TCGA-LIHC dataset was obtained form the UCSC Xena browser (Supplementary Tables 2 and 3).

To investigate the regulatory mechanism underlying CPAP-mediated enhancement of liver inflammation, reporter assay and WB analysis were performed to assess the activation of STAT3 and NF-κB in GFP-CPAP-overexpressing and CPAP-knock down hepatocytes. As shown in Fig. 5A, B, ectopic expression of CPAP increased the phosphorylation and transcriptional activity of STAT3 and NF-κB (Fig. 5A, B), whereas knockdown of CPAP inhibited the phosphorylation of STAT3 and NF-κB in hepatocytes (Fig. 5C). HA-CPAP overexpression increased *TNF-α* and *IL-8* gene expression (Fig. 5D); in contrast, knockdown of CPAP decreased *TNF-α* and *IL-8* expression (Fig. 5E). The association between CPAP and liver inflammation was evaluated in the NCKUH cohort (Supplementary Table 1) and The Cancer Genome Altas-Liver Hepatocellular Carcinoma (TCGA-LIHC) dataset (Supplementary Tables 2 and 3) using the lymphocytic infiltration level (Supplementary Table 3) and Ishak staging system (Supplementary Tables 1 and 2), which is widely used to assess the stages of liver fibrosis [38]. Interestingly, the expression of *CPAP* mRNA and *TNF-α* mRNA in HCC-adjacent normal tissues (NTs) was positively correlated with an increased inflammatory status (with mild and severe lymphocyte infiltration) (Fig. 5F, G), but was not correlated with the Ishak fibrosis scores (Fig. 5H, I, Supplementary Fig. S5). *CPAP* mRNA expression is positively correlated with *TNF-α* mRNA expression in HCC adjacent normal tissues (NTs) with mild and severe grades of lymphocytic infiltration (Fig. 5J, K). These results suggested that CPAP can induce chronic inflammation in the liver by increasing lymphocytic infiltration, which leads to hepatocarcinogenesis.

### Liver-secreted CCL-16 is a potential biomarker for chronic inflammation

In 2019, Andras Franko et al., proposed a secretome profile in primary human hepatocytes (PHHs) and HepG2 HCC cells via liquid chromatography with tandem mass spectrometry (LC-MS/MS) analysis [39]. A total of 691 and 745 secreted proteins were identified in PHHs and HepG2 cells, respectively. After performing a pathway enrichment analysis with g:Profiler (https://biit.cs.ut.ee/gprofiler), several HCC-specific expressed proteins were identified and further filtered by analysis of the Human Protein Atlas (https://www.proteinatlas.org/) (Supplementary Fig. S6). Three liver-specific secreted proteins potentially involved in the inflammatory response were identified: prothrombin (F2), which is highly expressed in HepG2 cells (HepG2 riBAQ [%] / PHH riBAQ [%] = 4.2); mannose binding lectin 2 (MBL2), which has a low expression level in HepG2 cells (HepG2 riBAQ [%] / PHH riBAQ [%] = 0.034); and CCL-16, which is expressed only in HepG2 cells (Supplementary Fig. S6). We first analyzed the expression of these three genes in the TCGA-LIHC dataset; surprisingly, we did not find overexpression of *CCL-16*, *MBL2*, or *F2* mRNAs in HCC tumor tissues (Supplementary Fig. S7).

**Fig. 6:**
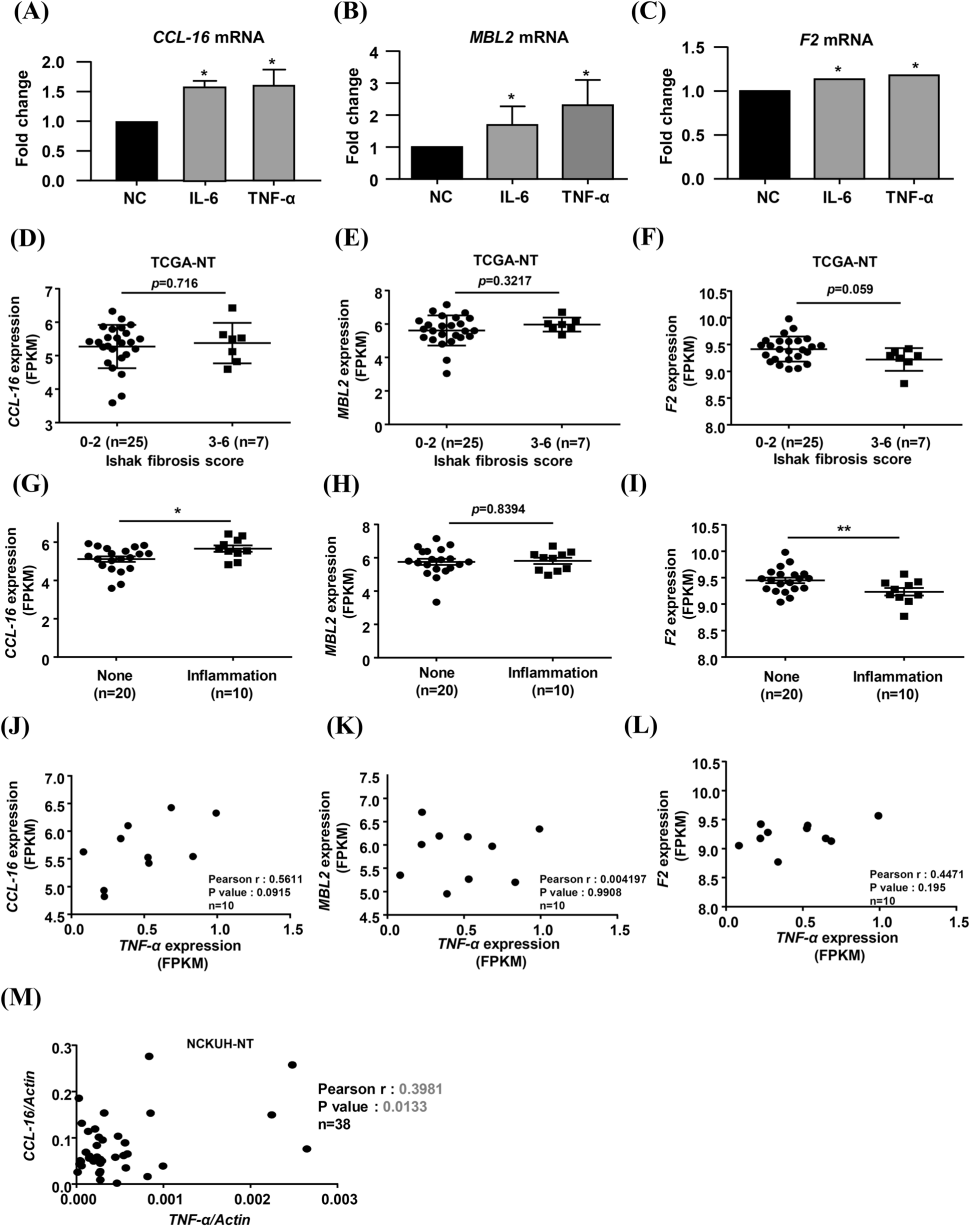
Liver-secreted CCL-16 is positively correlated with *TNF-α* expression. **A**–**C** The expression levels of *CCL-16*, *MBL2*, and *F2* mRNAs in human hepatocytes treated without (NC) or with IL-6 or TNF-α were determined by RT-qPCR. **D**–**I** The expression levels of *CCL-16*, *MBL2*, and *F2* mRNAs in adjacent normal liver tissues (NT) of HCC patients from the TCGA-LIHC dataset with different Ishak fibrosis scores (**D**–**F**) and lymphocytic inflammation statuses (**G**–**I**) are shown. **J**–**L** Correlations between *CCL-16*, *MBL2*, and *F2* mRNA levels and the *TNF-α* mRNA level in adjacent normal liver tissues with lymphocytic inflammation (*n* = 10) from the TCGA-LIHC dataset were identified by the Pearson correlation coefficients. **M** The correlation between *TNF-α* and *CCL-16* mRNA expression levels in the adjacent normal liver tissues of HCC patients (*n* = 38) from the NCKUH cohort was identified by the Pearson correlation coefficient. *p < 0.05, **p < 0.01.

We confirmed whether the expression of these liver-enriched genes is regulated by inflammatory signaling pathways in IL-6- or TNF-α-treated human hepatocytes. *CCL-16* and *MBL2* mRNA levels were increased in IL-6- or TNF-α-treated hepatocytes (Fig. 6A, B), and the *F2* mRNA level was slightly increased upon IL-6- or TNF-α-treatment (Fig. 6C). Moreover, the expression of *CCL-16*, *MBL2*, and *F2* mRNA weas not correlated with the Ishak fibrosis score in HCC-adjacent normal tissues (Fig. 6D–F). The *CCL-16* mRNA level was increased in HCC-adjacent normal tissues with lymphocytic infiltration, whereas the *F2* mRNA level was decreased in the same tissues (Fig. 6G–I). Only the *CCL-16* mRNA level had a positive correlation with the *TNF-α* mRNA level in HCC-adjacent normal tissues with lymphocytic infiltration (Fig. 6J–M); no correlation was observed between the mRNA expression levels of *MBL2* and *TNF-α* or *F2* and *TNF-α* (Fig. 6K, L). Studied have reported that the liver-specific chemokine CCL-16 can attract monocytes, lymphocytes and dendritic cells [18, 20]; in addition, a high concentration of CCL-16 in plasma can modulate inflammatory responses [16]. Our results suggested that CCL-16 could be a biomarker for the early detection of chronic liver inflammation.

**Fig. 7:**
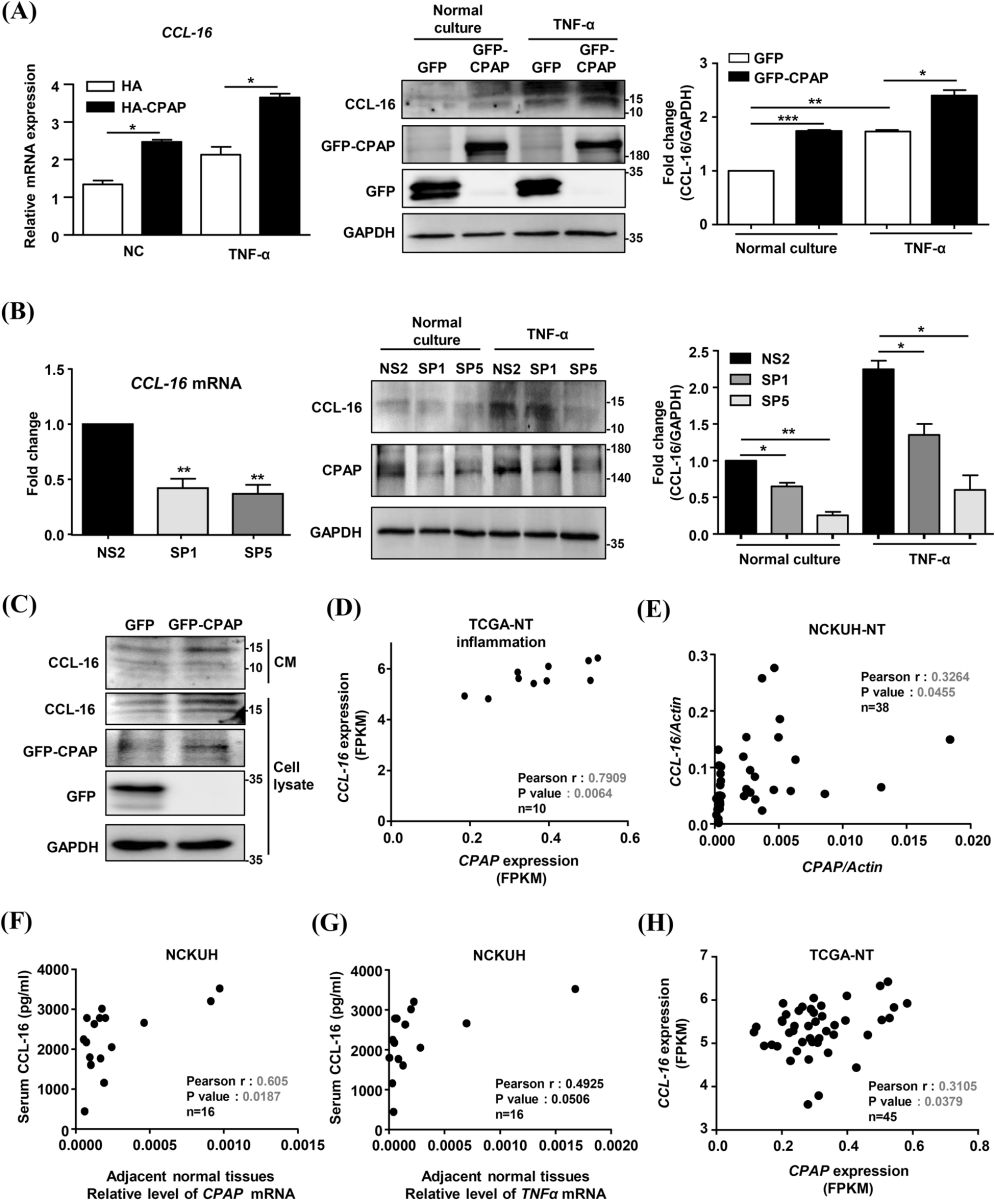
CPAP promotes inflammatory cell infiltration by increasing TNF-α/ CCL-16 signaling. **A** Human hepatocytes were transfected with HA and HA-CPAP (left), or with GFP and GFP-CPAP (right), and the expression of CCL-16 was then evaluated by RT-qPCR analysis (left) and WB analysis (right) under normal culture (NC) condition or after treatment with 10 ng/ml TNF-α for 24 h. **B** Human hepatocytes transfected with *CPAP* shRNA (SP1 and SP5) were collected to evaluate the expression of CCL-16 as described above. pSUPER-NS2 (NS2) is the siRNA vector control. The protein expression levels of CCL-16 are displayed as ratios and are shown below the blot image. **C** Conditioned media from GFP- and GFP-CPAP-transfected hepatocytes were collected to detect secreted CCL-16 by WB analysis. Total cell lysates were collected to determine the expression of GFP and GFP-CPAP. GAPDH was used as the loading control. The data are presented as the mean ± SEM values. *p < 0.05, **p < 0.01. **D**, **E** The mRNA expression levels of *CCL-16* and *CPAP* in adjacent normal liver tissues with lymphocytic inflammation were measured to determine their correlation by Pearson correlation analysis. TCGA-LICH dataset (**D**, *n* = 10): R = 0.7909, *p* = 0.0064; NCKUH cohort (**E**, n-38): R = 0.3264, *p* = 0.0455. **F**–**G** The levels of secreted CCL-16 protein, *CPAP* mRNA and *TNF-α* mRNA were determined in serum and adjacent normal liver tissues from the NCKUH cohort (*n* = 16). The correlations between *CCL-16* mRNA and *CPAP* mRNA levels (**F**) and between *CCL-16* mRNA and *TNF-α* mRNA levels (**G**) were assessed by the Pearson correlation coefficients. **H** The correlation between *CCL-16* mRNA and *CPAP* mRNA in the nontumoral part of HCC from the NCKUH cohort (*n* = 43).

Since CPAP plays an important role in inflammatory pathways in human hepatocytes, we investigated the effect of CPAP on CCL-16 expression in human hepatocytes. Overexpression of CPAP increased *CCL-16* expression in hepatocytes under both normal culture and TNF-α treatment conditions (Fig. 7A), whereas knockdown of *CPAP* decreased *CCL-16* expression (Fig. 7B). Additionally, GFP-CPAP overexpression increased the secretion of CCL-16 in hepatocytes under normal culture condition (Fig. 7C). The expression of *CCL-16* mRNA was positively correlated with that of *CPAP* mRNA in HCC-adjacent normal tissues with lymphocytic infiltration (Fig. 7D, E). In patients with HCC, serum levels of CCL-16 were positively correlated with the *CPAP* and *TNF-α* mRNA expression levels in HCC-adjacent normal tissues (Fig. 7F, G). The results from TCGA-LIHC dataset analysis showed that the mRNA expression levels of *CPAP* and *CCL-16* is positively correlated in the nontumoral part of HCC (Fig. 7H). These results suggested that CPAP can upregulate CCL-16 expression via the TNF-α/NF-κB pathway in inflamed hepatocytes and HCC microenvironment.

## Discussion

**Fig. 8:**
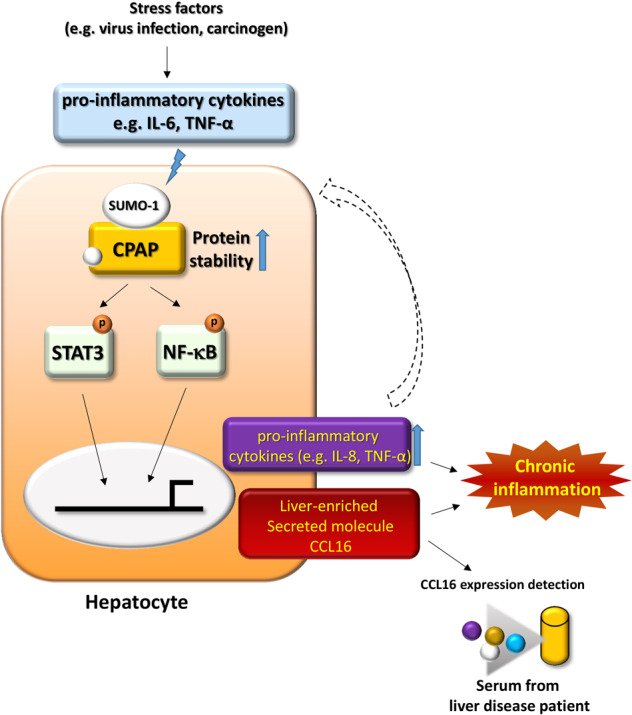
CPAP promotes and maintains liver inflammation by increasing the secretion of inflammatory cytokines via STAT3 and NF-κB. Upon viral infection or hepatic injury, human hepatocytes are under the stimulation of inflammatory cytokines, which increases CPAP protein stability through SUMO-1 modification. SUMO-1-modified CPAP enhances the expression of inflammatory cytokines, including TNF-α, IL-8, and CCL-16, by promoting the transcriptional activities of STAT3 and NF-κB. This CPAP-mediated enhancement of cytokine expression contributes to the chronic inflammatory microenvironment surrounding hepatocytes.

Inflammation is tightly associated with cancer development [40, 41]. Chronic inflammation and the inflammatory microenvironment of hepatocytes are believed to play a vital role in HCC progression [7]. Our results showed that overexpressed CPAP increases the expression of proinflammatory cytokines, including TNF-α, IL-6, IL-1β, IL-8, and liver-enriched CCL-16, by promoting the activation of two important inflammatory transcriptional factors—STAT3 and NF-κB—to create and maintain a chronic inflammatory microenvironment that facilitates the development of HCC (Fig. 8). In this study, we demonstrated that overexpressed CPAP enhances the inflammatory response by increasing the expression of proinflammatory factors TNFα, IL8, and CCL-16 in hepatocytes. By this finding, we suggest that serum CCL-16 levels might be used as a biomarker for early prediction of HCC onset; people with a high risk of HCC might be monitored by measuring the serum expression level of CCL-16 for early diagnosis.

Our results showed an obvious increase of CPAP protein levels in IL-6- and TNFα-treated human hepatocytes, whereas *CPAP* mRNA levels are not changed under the same treatment (Fig. 4B). However, in the mouse model, the *cpap* mRNA level was increased in the livers of DEN-treated mice (Fig. 4A). This differential expression pattern of *CPAP* mRNA may be due to the different treatments. DEN is widely used to induce hepatocarcinogenesis via the formation of DNA alkylation adducts after bioactivation by hepatic cytochrome P450 (CYP) enzymes [42]. Although our results indicated that DEN treatment enhances the expression of IL-6 and TNF-α (Fig. 2C), both of which can in turn increase the protein expression level of CPAP as observed in IL-6- and TNF-α-treated human hepatocytes (Fig. 4E, F); however, the mechanisms of DEN-increased *cpap* mRNA expression in the mouse liver remain unclear. One recent report indicated that DEN treatment can increase the expression of the transcription factor Yes-associated protein/transcriptional coactivator with PDZ-binding motif (YAP/TAZ) [43], an important transcription factor in HCC development [44]. Our previous report indicated that *CPAP* mRNA levels are increased in HCC [31]; in addition, we showed that the transcription factor CREB can upregulate *CPAP* expression in HCC [30]. In this study, our results showed that the increased CPAP expression in IL-6- and TNFα-treated human hepatocytes is mediated through SUMO-1 modification (Fig. 4E, F); our previous reports showed that the expression of *CPAP* mRNA increases during HCC progression [30, 31]. Therefore, the increased *cpap* mRNA level in the livers of DEN-treated mice may be due to unidentified transcription factors activated by DEN. The molecular mechanism of DEN-increased *cpap* mRNA expression needs further investigation.

In this study, the physiological correlation between CPAP overexpression and liver inflammation in human specimens was analyzed using two different categories, the Ishak fibrosis score and the lymphocytic infiltration level [38]. CPAP expression was not correlated with the Ishak fibrosis score in either the TCGA database or the NCKUH cohort, but was positively associated with the lymphocytic infiltration level (Fig. 5F–I). Although the *CPAP* Tg mice exhibited increased liver inflammation and some developed HCC (Fig. 1), no fibrosis was observed. In addition, in human specimens, the expression of CCL-16, which was increased upon IL-6 and TNF-α treatment (Fig. 6A), was positively associated with the inflammation level but not the Ishak fibrosis score (Fig. 6D-G). The positive correlations between CPAP and TNF-α (Fig. 5J), TNF-α and CCL-16 (Figs. 6J, M and 7G), and CPAP and CCL-16 (Fig. 7D-F) suggested that CPAP overexpression in hepatocytes is closely related to liver inflammation. Injured liver cells can release factors to attract immune cells for infiltration, and these immune cells then secrete chemokines to create an inflammatory microenvironment and further increase inflammatory cell infiltration [45]. Because there is no active Ccl-16 in mice [46, 47], we therefore cannot test the effects of Ccl-16 in *CPAP* Tg mice. However, by using human hepatocytes and clinical analysis, our results provided evidence to propose an additional liver inflammatory mechanism acting by overexpressed CPAP. We propose that overexpression of CPAP increases the level of CCL-16 to interact with its receptor CCR2, which is located in Kupffer cells and other recruited macrophages; these macrophages then secrete cytokines to attract more inflammatory cell infiltrating, in turn creating and maintaining an inflammatory microenvironment in liver tissues. The increased expression of CCL-16 could be used as a biomarker for early prediction of HCC development, and this is not correlated with liver fibrosis. More investigation is needed to further confirm the importance of CCL-16 in inflammation-induced hepatocarcinogenesis.

Currently, HCC therapy remains unsatisfactory. The response to small molecule inhibitors, such as sorafenib, regorafenib, lenvatinib, and the 5-fluorouracil, leucovorin, and oxaliplatin (FOLFOX4) regimen recently recommended by the FDA, is limited and accompanied by a high recurrence rate [48]. Recently, immune checkpoint blockade has provided an effective and promising therapeutic strategy for cancer [49]; the considerable anticancer effect of immune checkpoint inhibitors has been approved in a variety of cancers, including HCC [48]. Immunotherapy will undoubtedly become a promising therapeutic strategy for HCC. CCL-16 has been reported to be overexpressed in human hepatocytes and HepG2 cells [16], and the current studies suggest a potential role of overexpressed CCL-16 in liver inflammation which is important for the development of hepatitis and HCC. CCL-16 overexpression was positively correlated with liver inflammation (Fig. 6G), and the expression of CCL-16 was increased in inflamed hepatocytes in CPAP-overexpressing cells via enhancement of STAT3 and NF-κB activity (Figs. 6A and 7). Blockade of the pathways involved in CPAP-mediated CCL-16 expression or inhibition of the interaction between CCL-16 and its receptors may provide a novel strategy in hepatitis and HCC therapies. However, the anticancer effects of STAT3 and NF-κB inhibitors are limited, and these drugs may produce several side effects after long-term treatment [50, 51]; therefore, the usage of anti-CCL-16 antibodies to block the interaction of CCL-16 with its receptors may become an attractive strategy to block the interaction between CCL-16 and its receptors in treating hepatitis, preventing liver inflammation and HCC development.

## Materials and methods

### Establishment of Tg mice with liver-specific expression of CPAP

The *CPAP* gene was cloned by the SalI and PmeI in pAlb-In-pA-HS4 [32], under the control of a hepatocyte-specific *albumin* promoter. C57BL/6 J mice were used to generate *CPAP* Tg mice by pronucleus microinjection into fertilized eggs. The tails of individual 7-day-old mice were cut, and genomic DNA was isolated with a Mouse Direct PCR Kit (Biotool, Houston, TX, USA). The genotype of Tg mice was determined by PCR with the indicated primer pairs (please see Supplementary Fig. S1 and Supplementary Table 4). PCR amplification was performed under the following conditions: predenaturation at 94 °C for 5 min; 35 cycles of denaturation at 94 °C for 30 sec, annealing at 58 °C for 30 sec, and extension at 72 °C for 30 sec; a final extension step at 72 °C for 5 min; and holding at 4 °C. All mice were bred in a specific pathogen-free room. *HBx* Tg mice [32] were used to generate *CPAP/HBx* double-transgenic (CH Tg) mice. The animal experiments were approved by the Institutional Animal Care and Use Committee (IACUC104056) at National Cheng-Kung University.

### Determination of serum ALT/GPT activity

Mouse serum was collected to determine the ALT level with FUJI DRI-CHEM 4000i chemistry analyzers (FUJIFILM Corporation, Tokyo, Japan).

### Establishment of the DEN-induced acute hepatic injury and HCC mouse models

To establish the short-term DEN-induced acute hepatic injury model, two-week-old mice were injected intraperitoneally (IP) with 100 mg/kg DEN (N0756, Sigma-Aldrich, St. Louis, MO, USA) for 0, 4, 24 and 48 h; mice were sacrificed 48 h after injection. To establish the long-term DEN-induced HCC model, 15-day-old mice were injected IP with 25 mg/kg DEN; then the hepatic injury and hepatic tumorigenesis were monitored by ALT level measurement and ultrasound begining 5 months after DEN injection. Mice were sacrificed 9 and 11 months after DEN injection.

### Cell culture, transfection and reagents

Human hepatocytes (HHs) were purchased from ScienCell (HHs, Cat#5200, ScienCell Research Laboratories, Carlsbad, CA, USA), and cultured according to the manufacturer’s instructions. In brief, HHs were cultured in hepatocyte medium (HM, Cat#5201, ScienCell) supplemented with hepatocyte growth supplement (HGS, Cat #5252, ScienCell), 5% fetal bovine serum (FBS, Cat #0025, ScienCell) and 100 unit/ml penicillin/streptomycin (P/S, Cat#0503, ScienCell). HHs were transfected with plasmids as indicated in the text using PolyJet™ (SL100688, SignaGen, Rockville, MD, USA). Cells were treated with 25 ng/ml human IL-6 (GF338, Millipore, Billerica, MA, USA) or 10 ng/ml human TNF-α (GF314, Millipore, Billerica, MA, USA) after 18–20 h starvation in serum-free medium. All experiments were performed by three separate batches of HH. Cells were kept in mycoplasma-free by checking the mycoplasma contamination status once per month.

### Western blot (WB) analysis

Total cell lysates were harvested using RIPA buffer (50 mM Tris-HCl (pH 7.4), 150 mM NaCl, 1 mM EDTA, 1% Nonidet P-40, and 0.25% sodium deoxycholate) with protease inhibitor cocktails (Cat#P8340, Sigma-Aldrich, St. Louis, MO, USA). The antibodies used in this study are described below: anti-GFP (JL-8, Clontech, Mountain View, CA, USA), anti-CPAP (ab221134, Abcam, Danvers, MA, USA), anti-STAT3 (9139, Cell signaling, Danvers, MA, USA), anti-phospho-STAT3/Y705 (9145, Cell Signaling), anti-phospho-p65 (3036, Cell Signaling), anti-p65 (8242, Cell Signaling), anti-cleaved caspase-3 (9661, Cell Signaling), anti-GAPDH (sc32233, Santa Cruz, Dallas, TX) and anti-α-tubulin (DM1A, T6299, Sigma-Aldrich).

### RNA extraction, reverse transcription (RT) and quantitative PCR (qPCR)

Total RNA was extracted and purified as previously described [29]. RNA from tissues and cells was isolated using TRIsure™ reagent following the manufacturer’s instructions (BIO-38033, Bioline, London, UK). One microgram of RNA was reverse-transcribed with a High-Capacity cDNA Reverse Transcription Kit (Applied Biosystems) according to the manufacturer’s instructions. SYBR^®^ Green Supermix (170–8882, BIO-RAD, Hercules, CA, USA) was used for qPCR. Primer information is listed in Supplementary Table 4.

### STAT3 and NF-κB reporter activity assay

Cells were cotransfected with the STAT3 or NF-κB-driven reporter plasmid and the Renilla luciferase reporter plasmid using PolyJet™. The transcriptional activity of STAT3 or NF-κB was measured using a Dual-Luciferase Reporter kit (Promega, Madison, WI, USA) according to the manufacturer’s instructions.

### In situ proximity ligation assay (PLA)

Cells seeded on a sterile 12-mm coverslip were treated with 25 ng/ml IL-6 or 10 ng/ml TNF-α for 4 h and were then fixed with ice-cold EtOH/acetone (v/v 1:1) at −20 °C for 20 min. In situ PLA was performed as previously described [29] (Olink Bioscience, Uppsala, Sweden). Two primary antibodies derived from different species were used to recognize SUMO-1 (33–2400, Thermo Fisher Scientific, Inc., Waltham, MA, USA) and CPAP. The antibodies used in this study were anti-SUMO1 monoclonal antibody and anti-CPAP polyclonal antibody [52]. The bright red fluorescent puncta were amplified when a direct protein-protein interaction was present and detected using a fluorescence microscope (BX51, Olympus, Japan).

### HCC patient specimens

HCC specimens from National Cheng Kung University Hospital (NCKUH) were collected for this study with the approval of the Institutional Review Board (IRB), NCKUH, Tainan, Taiwan (B-ER-104–245). Studies involving clinical specimens were conducted in accordance with the 1975 *Declaration of Helsinki*, as revised in 1983.

### Bioinformatic and statistical analyses

Information on adjacent normal tissues with lymphocytic inflammation status and Ishak fibrosis scores, as well as mRNA expression data (normalized read counts of samples as FPKM values), was obtained from the UCSC Xena Browser (https://xenabrowser.net/). Parametric unpaired and paired two-tailed Student’s *t* tests were used to evaluate the differences between the control and experimental groups. Pearson correlation analysis was applied to analyze genes expression correlations. Data are presented as the mean ± SEM values. *p < 0.05; **p < 0.01; and ***p < 0.001.

## Supplementary information

Supplementary Figures

Supplementary Figure Legends

Supplementary Table 1

Supplementary Table 2

Supplementary Table 3

Supplementary Table 4

Reproducibility checklist

## Data Availability

The authors declare that all data supporting the findings of this study are available within the article.
