# Supplementary material for: CPAP enhances and maintains chronic inflammation in hepatocytes to promote hepatocarcinogenesis
Source: Cell Death Dis. 2021 Oct 22;12(11):983. doi: 10.1038/s41419-021-04295-2 (PMC8536685; doi:10.1038/s41419-021-04295-2)

Supplementary Figure S1

(A)

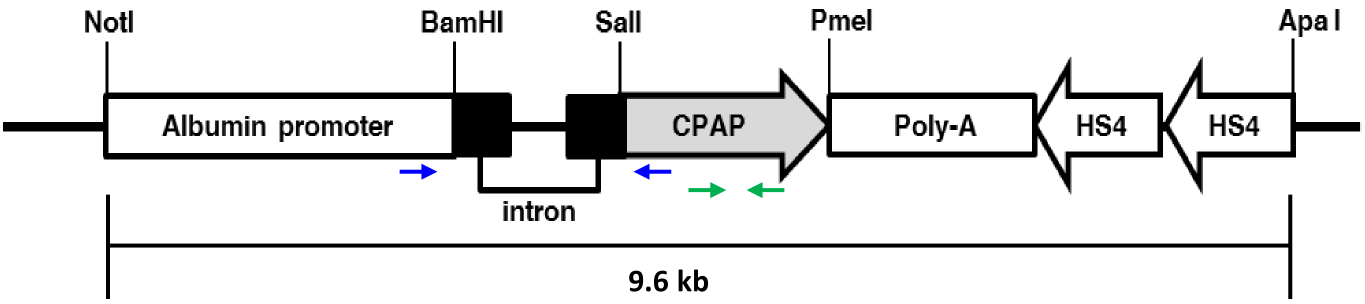

(B)

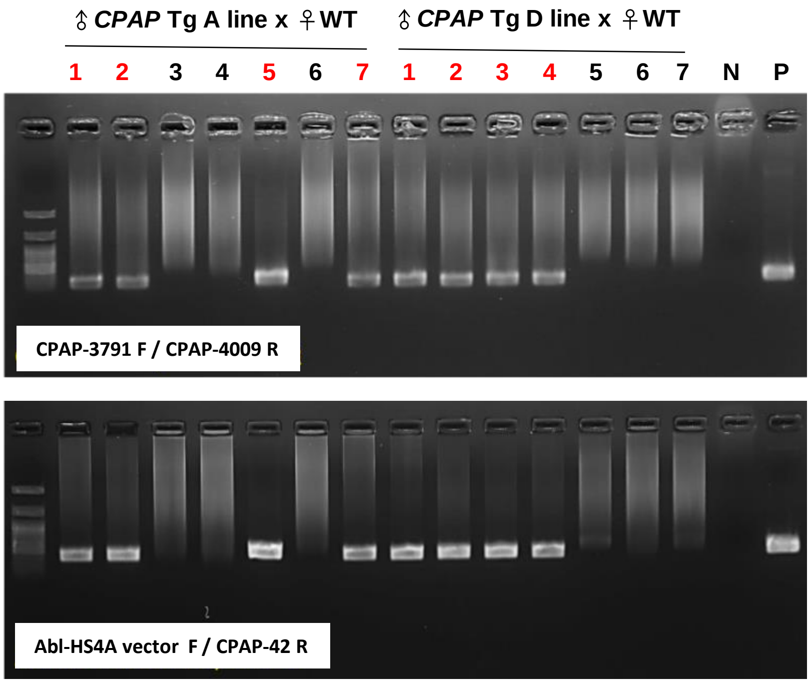

Supplementary Figure S2

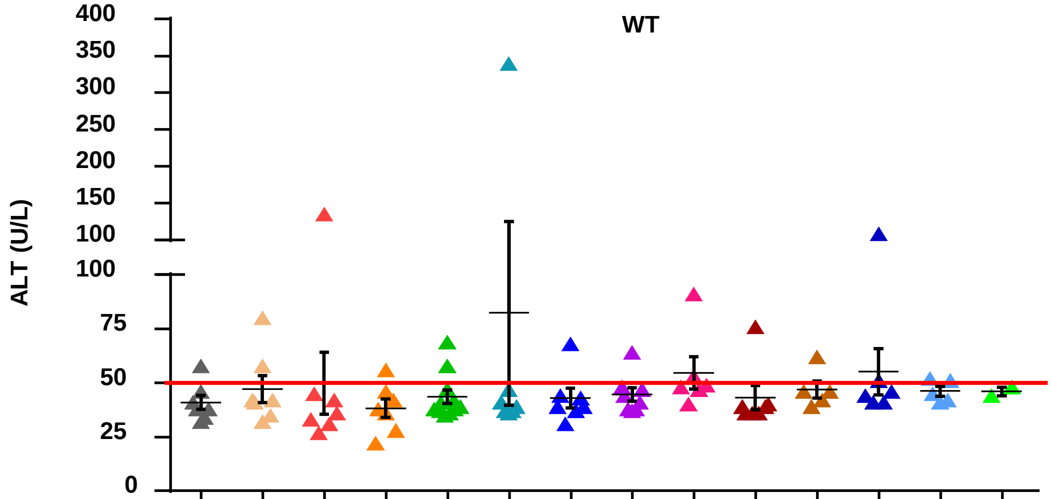

Age (months)	9	11	12	13	14	15	16	17	18	19	20	21	22	23
Number	1	2	1	1	2	1	1	1	2	1	1	2	2	0

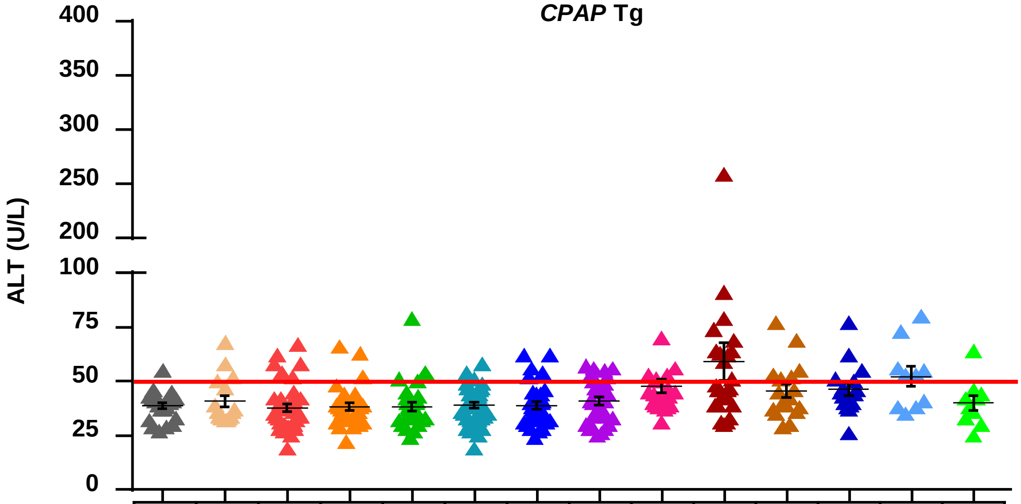

Age (months)	9	11	12	13	14	15	16	17	18	19	20	21	22	23
Number	1	4	6	3	4	3	5	6	7	11	6	5	6	1

Supplementary Figure S3

(A)

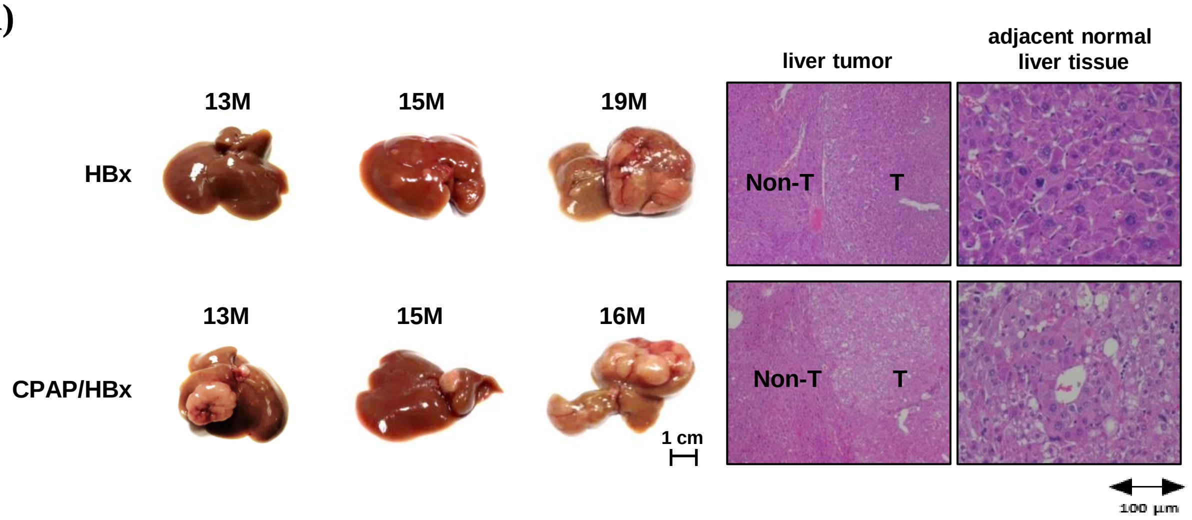

(B)

Number of bred mice developing HCCs per month interval						
Age in month	10-12	13-14	15-16	17-18	19-20	21-24
HBx	3/5 (60%)	3/5 (60%)	4/4 (100%)	2/2 (100%)	5/5 (100%)	1/1 (100%)
CPAP/HBx	2/2 (100%)	5/5 (100%)	3/4 (75%)	2/3 (66.7%)	-	-

(C)

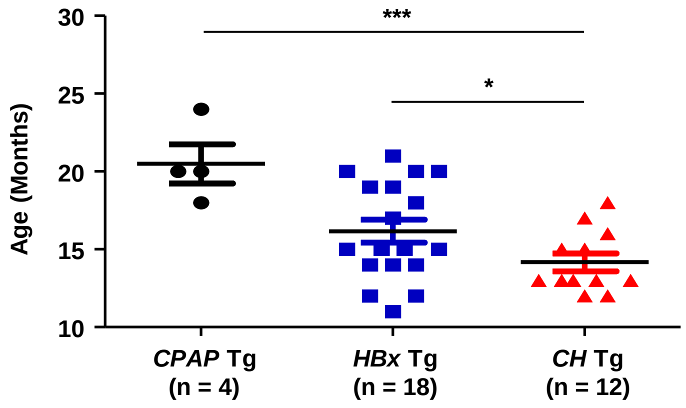

Supplementary Figure S4

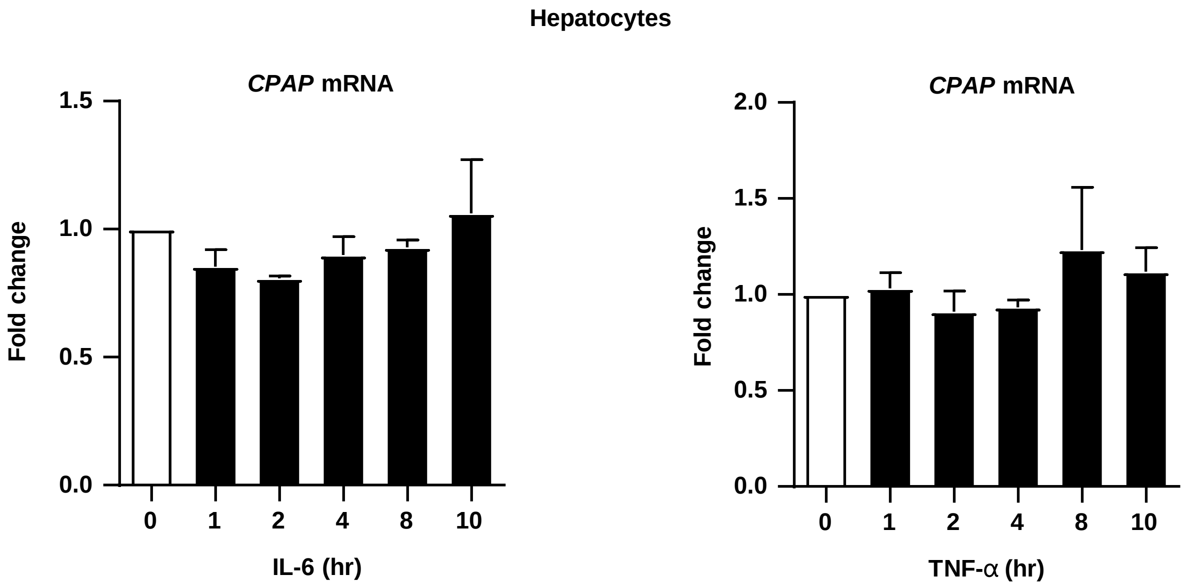

Supplementary Figure S5

(A)

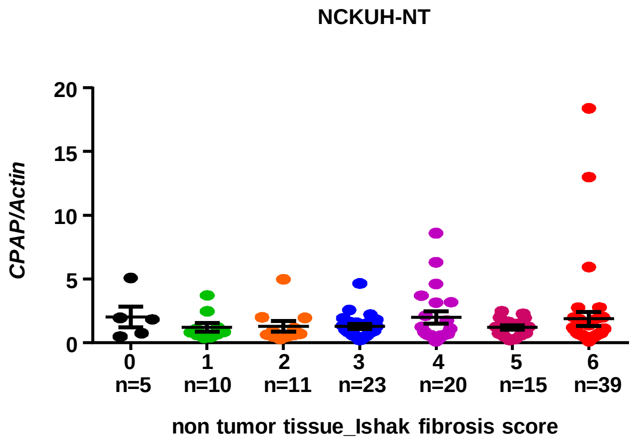

(B)

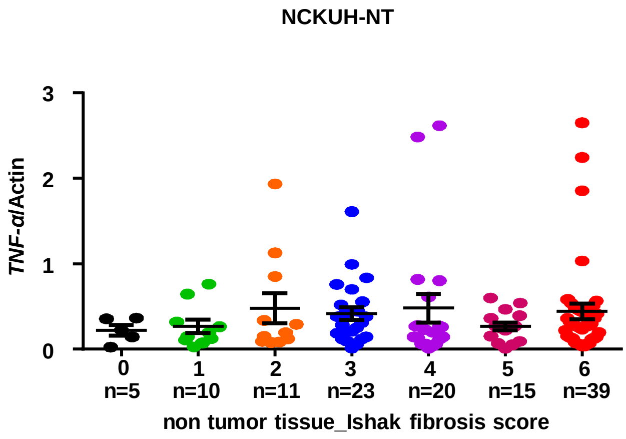

Supplementary Figure S6

Human hepatocytes | **HepG2**

LC-MS/MS analysis

predicting a signal peptide (SP+)

non-classical secretory proteins without signal peptide (SP−)

**Human hepatocytes**	**HepG2**
**691 secreted proteins**	**745 secreted proteins**
↓	↓
**389 SP+**	**477 SP+**

Pathway enrichment analysis :

g:Profiler - biological process gene ontology (GO) :

GO:0006954 **inflammatory response**

Only HepG2

HepG2 inflammatory response	HepG2 riBAQ [%]/PHH riBAQ [%]
IL1RAP	-
CCL16	-
ELANE	-
IL6ST	-
MDK	-
OSMR	-
PGLYRP2	-
PLA2G2A	-
SPP1	-

Up regualtion

HepG2 inflammatory response	HepG2 riBAQ [%]/PHH riBAQ [%]
APOA2	76.8
GPX4	45.9
AGT	28.7
C5	28.6
CHID1	26.6
LYZ	21.6
CX3CL1	13.3
SERPINF2	11.3
PROC	8.9
HYAL1	7.2
SERPINF1	6.9
APOA1	6.7
CTSC	6.3
CCL20	5.7
APOE	4.4
AHSG	4.3
F2	4.2

Down regulation

HepG2 inflammatory response	HepG2 riBAQ [%]/PHH riBAQ [%]
F12	0.034
MBL2	0.027
CXCL8	0.017
CD14	0.015
ITIH4	0.012
CHI3L1	0.003

Identification of **Liver-enriched secreted proteins** :

The human protein atlas database

↓	↓	↓
**CCL-16**	**Prothrombin (F2)**	**Mannose binding lectin 2 (MBL2)**

Supplementary Figure S7

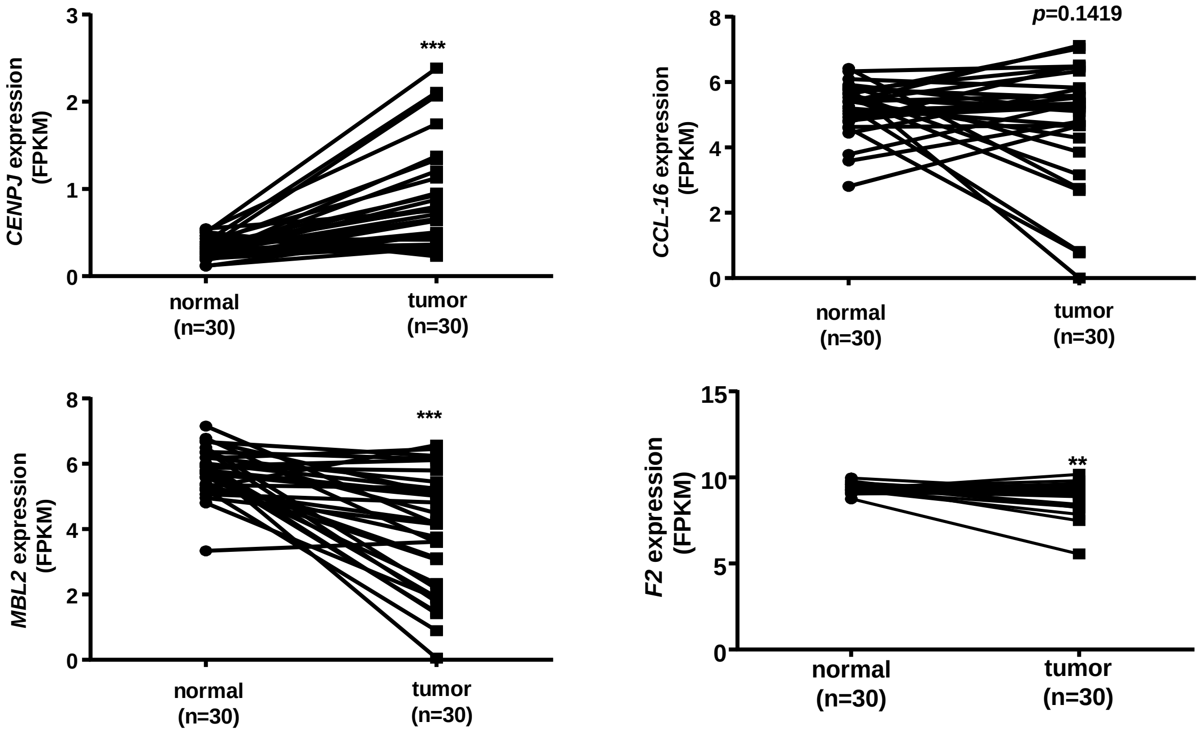

Supplement: Supplementary file 1 — Supplementary Figures [file 41419_2021_4295_MOESM1_ESM.pdf]
