# Supplementary material for: CPAP enhances and maintains chronic inflammation in hepatocytes to promote hepatocarcinogenesis
Source: Cell Death Dis. 2021 Oct 22;12(11):983. doi: 10.1038/s41419-021-04295-2 (PMC8536685; doi:10.1038/s41419-021-04295-2)
Supplement: Supplementary file 2 — Supplementary Figure Legends [file 41419_2021_4295_MOESM2_ESM.pdf]

Supplementary Figure S1. Establishment of the *CPAP* transgenic mouse. (A) Schematic illustration of the albumin-driven *CPAP* transgene. A 4014bp open reading fragment of *CPAP* was sub-cloned into the pAlb-In-pA-HS4 plasmid [1] through the SalI and PmeI sites. The *albumin* promoter-driven *CPAP* plasmid was identified by PCR analysis, enzyme digestion analysis and sequencing. **(B)** Heterozygous CPAP transgenic (*CPAP* Tg) mice were obtained by mating between *CPAP* heterozygotes and wild-type C57BL/6. The genotype of *CPAP* Tg mice were checked by PCR analysis at 7 days old using two pairs of primers—CPAP_3791F and CPAP_4219R (green color arrows in Figure S1A), Abl-HS4 vector_F and CPAP_42R (blue color arrows in Figure S1A).

Supplementary Figure S2. Serum ALT values of *CPAP* Tg and WT mice. Sera of *CPAP* Tg (lower) and WT (upper) mice in ages from 9 months to 23 months were collected to determine the ALT value. The number of mice with ALT values greater than 50 U/L is shown.

Supplementary Figure S3. *CPAP/HBx* double transgenic mice accelerate the development of HCC. (A) Gross images (left) and H&E stains (right) of mouse liver from *HBx* and *CPAP/HBx* transgenic (Tg) mice at different ages as indicated. Liver tumor and adjacent liver tissue are shown. T, tumor; Non-T, non-tumor. **(B)** The incidence of liver tumor in different ages of *HBX* Tg mice and *CPAP/HBX* Tg mice. **(C)** Ages of liver cancer incidence in *CPAP* (n = 4), *HBx* (n = 18) and *CPAP/HBx* Tg mice (n = 12).

Supplementary Figure S4. IL-6 and TNF-α treatment do not affect the expression of *CPAP* mRNA in hepatocytes. Human hepatocytes were treated with

25 ng/ml IL-6 (left) and 10 ng/ml TNF-α (right) for indicated time periods, and then the cells were collected to analyze the expression of *CPAP* mRNA by RT-qPCR.

Supplementary Figure S5. Expression of *CPAP* and *TNF-α* mRNAs is not correlated with fibrosis-induced inflammation in liver. The adjacent normal liver tissues (NT) of HCC with various Ishak fibrosis scores were collected from NCKUH to analyze the expression of **(A)** *CPAP* and **(B)** *TNF-α* mRNAs. 0=no fibrosis; 1=portal fibrosis (some); 2=portal fibrosis (most); 3=bridging fibrosis (occasional); 4=bridging fibrosis (marked); 5=incomplete cirrhosis; 6= cirrhosis.

Supplementary Figure S6. Overview of the analysis strategy for selecting potential secreted genes that bases on the results reported by Andras Franko et al. [2].

Supplementary Figure S7. Expression of *CPAP, CCL-16, MBL2,* and *F2* mRNAs in liver tumors and adjacent normal tissues. Analysis of C*ENPJ*, *F2*, *MBL2* and *CCL-16* expression levels in adjacent normal liver tissues and HCC tissues from the TCGA-LIHC dataset. The gene expression levels were normalized by the FPKM normalization method.

Reference

1. Wu, B.K., et al., *Blocking of G1/S transition and cell death in the regenerating liver of Hepatitis B virus X protein transgenic mice.* Biochem Biophys Res Commun, 2006. **340**(3): p. 916-28.
2. Franko, A., et al., *Identification of the Secreted Proteins Originated from Primary Human Hepatocytes and HepG2 Cells.* Nutrients, 2019. **11**(8).
