# Supplementary material for: CPAP enhances and maintains chronic inflammation in hepatocytes to promote hepatocarcinogenesis
Source: Cell Death Dis. 2021 Oct 22;12(11):983. doi: 10.1038/s41419-021-04295-2 (PMC8536685; doi:10.1038/s41419-021-04295-2)
Supplement: Supplementary file 3 — Supplementary Table 1 [file 41419_2021_4295_MOESM3_ESM.docx]

Supplementary Table 1. Ishak score of HCC adjacent hepatic tissues in NCKUH cohort.

| **Supplementary Table 1. NCKUH_adjacent_hepatic_tissues (fibrosis_ishak_score (n=123))** | |
| --- | --- |
| samples ID | fibrosis_ishak_score |
| 0497 | 6 |
| 0502 | 6 |
| 0486 | 6 |
| 0478 | 6 |
| 0444 | 6 |
| 0431 | 6 |
| 0398 | 6 |
| 0385 | 6 |
| 0367 | 6 |
| 0336 | 6 |
| 0331 | 6 |
| 0310 | 6 |
| 0274 | 6 |
| 0223 | 6 |
| 0221 | 6 |
| 0216 | 6 |
| 0201 | 6 |
| 0188 | 6 |
| 0182 | 6 |
| 0175 | 6 |
| 0150 | 6 |
| 0131 | 6 |
| 0113 | 6 |
| 0110 | 6 |
| 0101 | 6 |
| 0099 | 6 |
| 0059 | 6 |
| 0058 | 6 |
| 0049 | 6 |
| 0047 | 6 |
| 0042 | 6 |
| 0041 | 6 |
| 0030 | 6 |
| 0027 | 6 |
| 0024 | 6 |
| 0021 | 6 |
| 0018 | 6 |
| 0014 | 6 |
| 0001 | 6 |
| 0484 | 5 |
| 0465 | 5 |
| 0453 | 5 |
| 0389 | 5 |
| 0263 | 5 |
| 0256 | 5 |
| 0204 | 5 |
| 0143 | 5 |
| 0112 | 5 |
| 0069 | 5 |
| 0068 | 5 |
| 0037 | 5 |
| 0036 | 5 |
| 0015 | 5 |
| 0002 | 5 |
| 0492 | 4 |
| 0467 | 4 |
| 0462 | 4 |
| 0445 | 4 |
| 0435 | 4 |
| 0434 | 4 |
| 0427 | 4 |
| 0384 | 4 |
| 0314 | 4 |
| 0312 | 4 |
| 0278 | 4 |
| 0220 | 4 |
| 0210 | 4 |
| 0180 | 4 |
| 0145 | 4 |
| 0107 | 4 |
| 0073 | 4 |
| 0066 | 4 |
| 0048 | 4 |
| 0012 | 4 |
| 0479 | 3 |
| 0476 | 3 |
| 0468 | 3 |
| 0466 | 3 |
| 0451 | 3 |
| 0450 | 3 |
| 0438 | 3 |
| 0415 | 3 |
| 0396 | 3 |
| 0363 | 3 |
| 0224 | 3 |
| 0172 | 3 |
| 0151 | 3 |
| 0124 | 3 |
| 0120 | 3 |
| 0108 | 3 |
| 0097 | 3 |
| 0085 | 3 |
| 0084 | 3 |
| 0083 | 3 |
| 0052 | 3 |
| 0044 | 3 |
| 0013 | 3 |
| 0483 | 2 |
| 0342 | 2 |
| 0264 | 2 |
| 0199 | 2 |
| 0164 | 2 |
| 0116 | 2 |
| 0080 | 2 |
| 0076 | 2 |
| 0043 | 2 |
| 0032 | 2 |
| 0016 | 2 |
| 0461 | 1 |
| 0273 | 1 |
| 0257 | 1 |
| 0200 | 1 |
| 0178 | 1 |
| 0152 | 1 |
| 0081 | 1 |
| 0033 | 1 |
| 0031 | 1 |
| 0011 | 1 |
| 0209 | 0 |
| 0208 | 0 |
| 0197 | 0 |
| 0186 | 0 |
| 0166 | 0 |
