# Supplementary material for: CPAP enhances and maintains chronic inflammation in hepatocytes to promote hepatocarcinogenesis
Source: Cell Death Dis. 2021 Oct 22;12(11):983. doi: 10.1038/s41419-021-04295-2 (PMC8536685; doi:10.1038/s41419-021-04295-2)
Supplement: Supplementary file 4 — Supplementary Table 2 [file 41419_2021_4295_MOESM4_ESM.docx]

Supplementary Table 2. Ishak score ofHCC adjacent hepatitis tissues in TCGA_LIHC dataset.

| **Supplementary Table 2. TCGA_LIHC dataset (fibrosis_ishak_scor; n=32)** | |
| --- | --- |
| samples ID | fibrosis_ishak_score |
| TCGA-DD-A11B-11A | 6 - Established Cirrhosis |
| TCGA-DD-A11D-11A | 6 - Established Cirrhosis |
| TCGA-DD-A1EE-11A | 6 - Established Cirrhosis |
| TCGA-G3-A3CH-11A | 6 - Established Cirrhosis |
| TCGA-DD-A114-11A | 5 - Nodular Formation and Incomplete Cirrhosis |
| TCGA-BC-A10T-11A | 1,2 - Portal Fibrosis |
| TCGA-DD-A1EG-11A | 1,2 - Portal Fibrosis |
| TCGA-EP-A3RK-11A | 1,2 - Portal Fibrosis |
| TCGA-DD-A116-11A | 3,4 - Fibrous Speta |
| TCGA-DD-A1EH-11A | 3,4 - Fibrous Speta |
| TCGA-BC-A216-11A | 0 - No Fibrosis |
| TCGA-DD-A113-11A | 0 - No Fibrosis |
| TCGA-DD-A118-11A | 0 - No Fibrosis |
| TCGA-DD-A119-11A | 0 - No Fibrosis |
| TCGA-DD-A11A-11A | 0 - No Fibrosis |
| TCGA-DD-A11C-11A | 0 - No Fibrosis |
| TCGA-DD-A1EB-11A | 0 - No Fibrosis |
| TCGA-DD-A1EC-11A | 0 - No Fibrosis |
| TCGA-DD-A1EJ-11A | 0 - No Fibrosis |
| TCGA-DD-A1EL-11A | 0 - No Fibrosis |
| TCGA-DD-A39V-11A | 0 - No Fibrosis |
| TCGA-DD-A39X-11A | 0 - No Fibrosis |
| TCGA-DD-A3A1-11A | 0 - No Fibrosis |
| TCGA-DD-A3A2-11A | 0 - No Fibrosis |
| TCGA-DD-A3A3-11A | 0 - No Fibrosis |
| TCGA-DD-A3A4-11A | 0 - No Fibrosis |
| TCGA-DD-A3A5-11A | 0 - No Fibrosis |
| TCGA-DD-A3A6-11A | 0 - No Fibrosis |
| TCGA-DD-A3A8-11A | 0 - No Fibrosis |
| TCGA-EP-A26S-11A | 0 - No Fibrosis |
| TCGA-ES-A2HT-11A | 0 - No Fibrosis |
| TCGA-FV-A3I1-11A | 0 - No Fibrosis |
