# Supplementary material for: CPAP enhances and maintains chronic inflammation in hepatocytes to promote hepatocarcinogenesis
Source: Cell Death Dis. 2021 Oct 22;12(11):983. doi: 10.1038/s41419-021-04295-2 (PMC8536685; doi:10.1038/s41419-021-04295-2)
Supplement: Supplementary file 5 — Supplementary Table 3 [file 41419_2021_4295_MOESM5_ESM.docx]

Supplementary Table 3. Inflammation score of HCC adjacent hepatic tissues in TCGA_LIHC dataset.

| **Supplementary Table 3. TCGA_LIHC dataset**  **(adjacent_hepatic_tissue_inflammation_extent_type; n=30)** | |
| --- | --- |
| samples ID | inflammation score |
| TCGA-DD-A1EE-11A | Severe |
| TCGA-G3-A3CH-11A | Severe |
| TCGA-BC-A216-11A | Mild |
| TCGA-DD-A1EJ-11A | Mild |
| TCGA-DD-A1EL-11A | Mild |
| TCGA-EP-A26S-11A | Mild |
| TCGA-ES-A2HT-11A | Mild |
| TCGA-DD-A1EG-11A | Mild |
| TCGA-DD-A114-11A | Mild |
| TCGA-DD-A11B-11A | Mild |
| TCGA-DD-A113-11A | None |
| TCGA-DD-A118-11A | None |
| TCGA-DD-A119-11A | None |
| TCGA-DD-A11A-11A | None |
| TCGA-DD-A11C-11A | None |
| TCGA-DD-A1EB-11A | None |
| TCGA-DD-A1EC-11A | None |
| TCGA-DD-A39V-11A | None |
| TCGA-DD-A39X-11A | None |
| TCGA-DD-A3A1-11A | None |
| TCGA-DD-A3A2-11A | None |
| TCGA-DD-A3A3-11A | None |
| TCGA-DD-A3A4-11A | None |
| TCGA-DD-A3A5-11A | None |
| TCGA-DD-A3A6-11A | None |
| TCGA-DD-A3A8-11A | None |
| TCGA-DD-A1EH-11A | None |
| TCGA-DD-A11D-11A | None |
| TCGA-BC-A10X-11A | None |
| TCGA-BC-A10Z-11A | None |
