# Supplementary material for: CPAP enhances and maintains chronic inflammation in hepatocytes to promote hepatocarcinogenesis
Source: Cell Death Dis. 2021 Oct 22;12(11):983. doi: 10.1038/s41419-021-04295-2 (PMC8536685; doi:10.1038/s41419-021-04295-2)
Supplement: Supplementary file 6 — Supplementary Table 4 [file 41419_2021_4295_MOESM6_ESM.docx]

**Supplementary Table 4. Primers used in this study.**

| **qPCR Primers for Sybr green** | | |  |
| --- | --- | --- | --- |
| Gene | Forward primer (5’→3’) | Reverse primer (5’→3’) |  |
| **Human** |  |  |  |
| CPAP | GAAGGACAAGCTTGCGAACAC | GAGGTGGATTGCCTTGCATTG |  |
| TNF-α | CTTCTCCTTCCTGATCGTGG | GCTGGTTATCTCTCAGCTCCA |  |
| IL-8 | GACAAGAGCCAGGAAGAAACC | CTTTAGCACTCCTTGGCAAAA |  |
| VEGF | CTACCTCCACCATGCCAAGT | CCATGAACTTCACCACTTCGT |  |
| IL-1β | ATGGGATAACGAGGCTTATGTG | CAAGGCCACAGGTATTTTGTC |  |
| CCL16 | TTCGTCACCAAGAGGAACCG | CAAGTTCCTGGTAGGCAGCA |  |
| MBL2 | CCTGTGAGGATGCCCAAAAGAC | GTTCCCCCTTTTCTCCCTTGG |  |
| F2 | CGCTGGAGGACAAAACCGAAAG | CGGAAAAGCATCACCTGCCAAG |  |
| Actin | ATTGGCAATGAGCGGTTC | GGATGCCACAGGACTCCAT |  |
| **Mouse** |  |  |  |
| cpap | CTGCAGTGAGATTCCCCGTT | CATCAGGTGACCCTGAACCC |  |
| il-1β | CGGCACACCCACCCTG | AAACCGCTTTTCCATCTTCTTCT |  |
| tnf-α | CATCTTCTCAAAATTCGAGTGACAA | TGGGAGTAGACAAGGTACAACCC |  |
| actin | GGTACGACCAGAGGCATACAG | ACTGGGACGACATGGAGAAG |  |
|  |  |  |  |
| **qPCR Primers for TaqMan probe** | | | |
| Gene | Forward primer (5’→3’) | Reverse primer (5’→3’) | Probe |
| Human -IL-6 | ATCCTCGACGGCATCTCAG | GCTGCTTTCACACATGTTACTCTTG | CTGAGAAAGGAGACAT |
| Mouse-il-6 | TTCTCTGGGAAATCGTGGAAA | CAAGTGCATCATCGTTGTTCATAC | AGAGTTGTGCAATGG |
|  |  |  |  |
| **Sequence of primers used for genotping of CPAP tansgenic mice** | |  |  |
| CPAP 3791-F | ATGGTGACGTGAAGCAGGTCATGCC |  |  |
| CPAP 4009-R | CCGTGTCCATTGACACACCACCCTC |  |  |
| Abl-HS4A vector F | GAGTGACAATGACATCCACTTTGCC |  |  |
| CPAP R | GTCATCCACTGGGTTAGGAAGTTC |  |  |
